# Electroencephalogram (EEG) Alpha Power as a Marker of Visuospatial Attention Orienting and Suppression in Normoxia and Hypoxia. An Exploratory Study

**DOI:** 10.3390/brainsci10030140

**Published:** 2020-03-02

**Authors:** Alberto Zani, Clara Tumminelli, Alice Mado Proverbio

**Affiliations:** 1 School of Psychology, Vita e Salute San Raffaele University, 20132 Milan, Italy; 2 Institute of Molecular Bioimaging and Physiology (IBFM), National Research Council (CNR), 20090 Milan, Italy; 3 Dept. of Psychology, University of Milano-Bicocca, 20126 Milan, Italy; c.tumminelli@campus.unimib.it (C.T.); mado.proverbio@unimib.it (A.M.P.)

**Keywords:** EEG, FFT, alpha desynchronization, attention orienting, alerting, attention inhibition, neurocognitive perceptual and motor workload, hypoxia, overt motor responses, hemispheric lateralization

## Abstract

While electroencephalogram (EEG) alpha desynchronization has been related to anticipatory orienting of visuospatial attention, an increase in alpha power has been associated to its inhibition. A separate line of findings indicated that alpha is affected by a deficient oxygenation of the brain or hypoxia, although leaving unclear whether the latter increases or decreases alpha synchronization. Here, we carried out an exploratory study on these issues by monitoring attention alerting, orienting, and control networks functionality by means of EEG recorded both in normoxia and hypoxia in college students engaged in four attentional cue-target conditions induced by a redesigned Attention Network Test. Alpha power was computed through Fast Fourier Transform. Regardless of brain oxygenation condition, alpha desynchronization was the highest during exogenous, uncued orienting of spatial attention, the lowest during alerting but spatially unpredictable, cued exogenous orienting of attention, and of intermediate level during validly cued endogenous orienting of attention, no matter the motor response workload demanded by the latter, especially over the left hemisphere. Hypoxia induced an increase in alpha power over the right-sided occipital and parietal scalp areas independent of attention cueing and conflict conditions. All in all, these findings prove that attention orienting is undergirded by alpha desynchronization and that alpha right-sided synchronization in hypoxia might sub-serve either the effort to sustain attention over time or an overall suppression of attention networks functionality.

## 1. Introduction

The human brain continuously receives sensory and cognitive inputs, including even unimportant and unnecessary information for thriving and survival. Therefore, our attentional system must carry out a selection of the most relevant information to be thoroughly processed. Two different neural systems are involved in carrying out this selective function: An endogenous or voluntary system and an exogenous or automatic system [1,2]. Both hemodynamic and event-related potential (ERP) source neuroimaging studies identified in the frontal and in the temporo-parietal lobes the visual-spatial attentional endogenous and exogenous control areas [3,4], respectively. Corbetta and Shulman [3] and Shulman et al. [5] identified the endogenous attention system in the dorsal frontal-parietal areas. Indeed, both processes would primarily engage higher-level cortical circuits including, for endogenous orienting, frontal, parietal and temporal regions, and particularly the frontal eye fields (FEF) and the intraparietal sulcus (IPS) [6,7], and, for exogenous orienting, the right temporo-parietal junction (TPJ) [5,8]. Interestingly, the left frontal areas were more active than the parietal areas, but only during endogenous attention. In alerting tasks, endogenous and exogenous attention interact, and there is an increase both in global perceptual sensitivity and in the perception of exogenous spatial cues. Recently, Han and coworkers [9] showed that anterior insula (AI) is the key structure in endogenously reorienting of attention after reflexive attention is captured by a salient distractor. In tasks in which the participants know that a target will appear after a cue, brain responses will be optimized, and endogenous attention will be deployed [10]. There is an increased activation of the dorsal system when a stimulus appears at the same point in space of the cue. Top-down signals originate in the dorsal system and modulate sensory areas activity, detecting the current position of the presented stimuli [11]. Stimuli presented outside the attentional focus automatically catch attention and activate exogenous attention control in consequence of which ventral parietal areas and frontal cortex are activated. These stimuli activate the right-sided TPJ and the left-sided inferior frontal gyrus (IFG) [5]. Compared to exogenous spatial cues, vigilance tasks induce changes in endogenous and exogenous attention interactions and perceptual sensitivity. The sensitivity is reduced when stimulus onset is predicted, because an exogenous cue and the prediction time prepare participants to take over next stimulus [9]. Neurocognitive attentional processes related to the environmental input generate brain waves and electrical potentials with specific frequencies and amplitudes. In attentional processes, alpha band frequency, ranging between 7–14 Hz, plays a dominant role. Alpha electroencephalogram (EEG) activity is renown, like a typical response of the brain during relaxed wakefulness, for example, when the eyes are closed and/or attention is not heeded to any incoming information. Overall, this EEG frequency band has an occipito-parietal scalp distribution. It has been proposed that alpha oscillations provide pulsed inhibition for gamma activity and thereby dynamically route cortical information flow [12]. Kelly and colleagues [13,14] showed that alpha band activity was synchronized during visual selective or sustained attention tasks based on the presentation of visuospatial cues. The increase of this activity showed to be related to the inhibition of active processing of irrelevant and unexpected stimuli and contributed to the sustaining of top-down attentional control. Magnetoencephalography (MEG) studies (e.g., [15]) on visual attention perception also showed a pre-stimulus alpha activity. Most interestingly, a synchronization of alpha power in the parietal-occipital sulcus showed to be related to a functional modulation of information discrimination ability as undergirded by an overall inhibition of both the posterior occipital areas and the dorsal visual stream. This inhibition blocked out irrelevant information and avoided interference in working memory. Additionally, Foxe et al. [16] used an endogenous cueing task and found a different modulation of alpha rhythm at parietal-occipital areas. Furthermore, they also found that an alpha band desynchronization—or decrease—related to target information active processing possibly reflected a preparatory process [17], and that, during the valid visuospatial cueing task, an asymmetric, contralateral alpha deactivation in visual areas optimized orienting of visuospatial attention [18]. When a cue appeared prior of a target and the task required a greater attentional effort, Foxe and coauthors [16] also showed that alpha activity inhibited part of the visual space field in which interfering flankers were presented. Interestingly, alpha could be localized to the occipital-parietal areas of both hemispheres, and was active before the presentation of a stimulus, as if it had a role in the attentional preparatory mechanisms [18]. Indeed, while occipital-parietal areas integrate cue-related sensory information from multiple sensory modalities for programing the next engagement of visual attention, the inferior parietal cortex (IPC) would take care of maintaining attention orienting.

Experimental evidences have been provided showing task-related changes both in alpha power and scalp hemispheric lateralization or dominance. Indeed, a decrease in alpha power (i.e., alpha desynchronization) was found at occipital-parietal areas in attention orienting tasks, while an increase in this index was found during sustaining of attention [19]. Moreover, Li et al. [20] also found that task difficulty and visual stimulus handling could change alpha band amplitudes in different ways and at different points in time during endogenous processing. The participants to the study had to decide whether an object was a car or a face; alpha activity showed a higher amplitude at the right-sided occipital-parietal regions when the decisional task was more difficult. Volberg et al. [21] studied changes in alpha oscillatory activity in tasks of visual attention globally or locally directed by a cue. In this experiment, the subjects were prompted with a sound associated to the appearance of the target at a certain point of the screen, and with different sounds associated to the presence of the stimulus at any point of the screen. They saw that, in a task demanding a certain speed of response and in which the target was presented at a certain point of the screen, the right occipital-parietal area was activated for processing of global information, and, consequently, the left homologous region had a greater presence of alpha band, being inhibited. Conversely, the left hemisphere became more active in response to a target stimulus fallen at a specific location and the processing of its features at local level, so that the right hemisphere was in this case inhibited. Interestingly, alpha band was prevalent before the presentation of the stimulus, as if it had a role in the attention preparatory mechanisms. Alpha rhythm power measured at frontal-parietal areas during top-down processing was functionally interpreted as if the brain dealt with participants’ expectations towards the task. Most interestingly, alpha modulation in the posterior scalp areas is governed by the anterior, prefrontal areas, which have an important role in attentional selection: this might be a neural signature of executive control, in relation to which prefrontal and posterior occipital-parietal areas form an attention-related network. Wavelet-based EEG studies have also found an interaction between top-down and bottom-up processing hinting at the view that alpha activity preceding a stimulus would reflect a top-down preparatory mechanism modulating both the response timing and the performance in an attentional task [22].

In selective attention, alpha rhythm is higher on parietal-occipital sites. Furthermore, it has been proved that prefrontal cortex rules alpha power in posterior area during top-down processing. Wavelet Analysis studies also measured EEG oscillation bands in terms of spatio-temporal frequency. A desynchronization of alpha oscillations was related to a perceptual difficulty in identifying a visual target in a discrimination task (target/mask). Larger pre-stimulus desynchronizations showed to be closely related to a better performance and to anticipatory attention [23].

As far as brain frequency-specific oscillatory activity in relation to cognitive functions is concerned, it is an acknowledged assumption that alpha power is affected by its oxygen supply. Indeed, from an inadequate blood oxygen delivery to the brain, it may in fact derive hypoxia, which is a common feature in many clinical disorders, including severe anemia, respiratory diseases (e.g., serious asthma and sleep apnea), and ischemic brain lesions, with or without a coma. This condition often also occurs in healthy individuals faced with extreme operational environments, such as high-altitude (HA) and/or low air pressure milieus (e.g., acknowledged alpinists and climbers as well as HA natives), often in relation with very low temperatures.

Altered EEG recordings, considered by clinicians as indicators of cerebral metabolism and useful tools for evaluating hypoxia severity—i.e., hypoxia with or without ischemia—in individuals affected by such a condition (e.g., [24,25,26]), have generally been reported in many electrophysiological studies based on different types of both acute and chronic hypoxia. In human EEG studies of transient hypoxia, for instance, the latter was induced by low oxygen gas mixtures or hypoxic normobaric hypoxia (e.g., [27]), by simulated HA in hypobaric pressurized chambers (e.g., [28,29,30,31]), during rapid ascent to HA and lowland reoxygenation (e.g., [32,33,34]), and in HA natives [35]. Overall, a slowing activity of EEG in hypoxia with respect to normoxia has been generally advanced by all these studies.

As for the specific effects of acute hypoxia—regardless of the method of its induction—on spectral power density in the alpha frequency band, somehow inconsistent findings can be found in the literature. For example, 19 min of hypobaric hypoxia resulted in a significant decrease of alpha power (i.e., 8.9–11.8 Hz) in the spontaneous EEG of healthy volunteers as measured at the P_4_-O_2_ bipolar derivation [28]. Schellart and Reits [27] found that alpha content of brain spontaneous EEG during systemic hypoxia was strongly affected by the ‘‘eyes open/closed’’ volunteers’ condition. In contrast to the eyes closed condition, a transient increase of alpha synchronization occurred during the eyes-open condition. An alpha (i.e., 10–11 Hz) spectral power selective desynchronization at a reduced air pressure of 3000 m, and a meaningful gradual desynchronization of this frequency with increasing altitude to 4000, 5000, and 6000 m, respectively, was found in the posterior scalp areas by Ozaki et al. [29], in volunteers who did not perform any active task during EEG recordings. Conversely, Papadelis and coauthors [30] showed an absolute significant increase of alpha power during acute hypoxia as induced by decreasing the pressure of a barometric chamber, compared to 100% oxygen condition, in right-handed volunteers who performed a simple computerized flight-simulation task in which participants had to keep a continuously moving aircraft-target as much closer to the center of a personal computer (PC) screen denoted by a rectangle, in order to avoid the drifting of the target towards the edges of the screen if no control was applied [30]. Additionally, a significant increase of alpha synchronization at posterior scalp sites after an HA chronic acclimatization of 30 days, but not after a sojourn of seven days only, with respect to previous HA EEG baseline, together with a cogent desynchronization after re-descending to lowland, have instead been described by Zhao and coworkers [34], in the spontaneous EEG of a group of young soldiers who did not perform any tasks during the recording sessions.

Inasmuch, both alpha oscillatory synchronization or desynchronization have been associated with attention, attention-orienting inhibition, long-range synchronization, memory performance, and inhibition of interfering visual memories (e.g., [36,37]); these inconsistencies represent a nuisance for a more specific definition of both the functional significance of this EEG oscillatory activity in relation to attentional functions and, most importantly, of their possible alterations with hypoxia.

However, because attention is not a unitary function, it is important to relate EEG alpha oscillatory activity to the three specialized networks sub-serving the human attentional system as originally conceptualized by Posner and Petersen [38]. After the reviewing of many neuroanatomical and neuropsychological studies, in fact, Posner’s group [39], advanced the view that human attention system is undergirded by three different networks regulating different sub-processes, which is, alerting, orienting, and executive control. The alerting system would take care of achieving and maintaining an alert state; it would be focalized in frontal and parietal regions of the right hemisphere (RH). The orienting system, driving attentional focus on a specific point of space, would be localized in the frontal-parietal areas. Executive control, allowing to solve conflicting cognitive situations and psychomotor responses, would be associated with medial frontal regions and lateral prefrontal cortex.

To analyze the functional activation and interdependence/independence of these networks, Fan et al. [40] devised a so called Attention Network Test (ANT) based on the presentation of four different cueing conditions, that is, NC (no cue), CC (central cue), 2C (two vertically lateralized spatial cues at the same time above and below the fixation point), and LC (one vertically lateralized spatial cue above or below the fixation point). Each of these cueing conditions was randomly followed by directionally congruent and incongruent strings of five arrows, of which the central one, which might point either toward the left or to the right, posed as a target, and the peripheral ones as low-level (congruent condition) or high-level interfering (incongruent condition) flankers. A third target condition made up of a central arrow-target pointing either leftward or rightward surrounded by four simple straight-lines was originally included. Due to some inessential experimental findings and to the theoretical interpretation of the latter in several behavioral studies, Posner’s group later hemodynamic (i.e., functional magnetic resonance imaging (fMRI) [41]) and electrophysiological imaging (i.e., EEG [42]) studies omitted the 2C cue type and the target-arrow-surrounded by straight-line flankers condition, and used three cueing types, namely the NC, CC, and LC conditions, and two target-arrow vs. flanker-arrows conditions only.

### 1.1. ANT and Functional Meaning of its Cueing Conditions

The ANT has shown to be a useful and simple measure of attentional efficiency so that it can be used with adults, children, monkeys and patients with various abnormalities of attention [40]. As the cueing conditions are concerned, both neutral (CC) and valid spatial cues (LC) serve as a form of alerting cue, but only the latter provides always valid, predictive spatial information that allows participants to begin orienting attention to the appropriate location before the target is presented. Conversely, the central cue (CC) tends to keep reflexive attention focused on one location, which is the central location at the fixation cross, before the target exogenously attract orienting of attention at its appearance location. Unlike for these cueing conditions, under NC, there are neither alerting nor spatial cues and participants see only the fixation cross and the sequence of randomly presented targets above or below it. According to Fan et al. [40], the NC condition is a relatively low-alertness tonic orienting condition in which attention tends to remain diffused across the two potential target locations.

Since the three sources of attention, alerting, orienting, and executive attention appear to engage separate brain mechanisms, Fan et al. [40] investigated the independence and/or interaction of these networks and concluded that the latter were relatively independent mechanisms. Indeed, while phasic alerting enhanced flanker interference costs, both LC and NC conditions reduced them. According to Fan et al. [40], this occurred, in the first case, because participants directed attention to the target ahead of time, and, in the second case, because the NC condition is a relatively low-alertness condition and resulted in longer RTs and relatively lower errors. Consistent behavioral and electrophysiological findings were more recently found by Zani and Proverbio [43], in that both RTs and mean latency measures of a so-called ERPs conflict negativity (CN) component showed a decreased flanker conflict interference for both NC and LC cueing conditions as compared to both CC and 2C conditions. Overall, these findings support the view that, far from being independent from each other, the attentional system networks somehow directly interact one another.

### 1.2. Rationale for the Present Study

Purpose of the present study was to identify the most prominent features of EEG alpha power in the classification of attention modes and response control as related to the ANT neural networks with the hope of providing alpha-based reliable markers of their functionality. Moreover, we wanted to assess the possible impact of respiratory hypoxia on these three attention networks because not yet investigated and renown up to now. More specifically, we wanted to tap possible differences in this impact between conditions of lower or greater motor and cognitive workload. To achieve these aims we used the afore-mentioned ANT-SR (ANT-Slightly Redesigned) version and measured the decrease and/or increase of alpha power synchronization as a function of the possibly independent and/or partly interdependent activation of the three attention networks. We wondered whether hypoxia would have affected the three attentional networks independently from one another or in interaction.

Considered that ANT paradigm is an extremely simple task so that “The instructions to the subjects only require they know how to press a left key for a leftward-pointing arrow and a right key for a rightward-pointing arrow [40]”, we slightly redesigned it with the goal to carry out a behavioral and electrophysiological study aimed to find possible differences in the effects of hypoxia on a simple with respect to a difficult, psycho-motor overloaded attention orienting task. In details, we devised one cue-target condition in which a valid, spatially informative local cue preceded the delivery of one out of two different types of arrow-strings which had to be discriminated to provide a dual motor-choice overt response with the index or medium fingers of the left or right hand, depending on the type and direction of the arrowhead, no matter the congruency of the flankers. In this difficult motor variant, defined by us “LCmot”, the participants had to use one out of four fingers to give their response. The goal was to investigate the mechanisms of visuospatial attention, and, more specifically, of attentional conflict control situations during the execution of a relatively difficult motor task. In order to compare the effects of hypoxia on this condition with those possibly occurring in a simple orienting of attention condition, besides on alerting and executive conditions, we added to our ANT-SR the no Cue (NC), the Central Cue (CC), and the Local Cue (LC) conditions used in the original version of the ANT paradigm by Fan and colleagues [40], and in Fan’s et al. [41,42] later studies. However, to make the four cue conditions comparable from the stimulus-information point of view, the two types of arrows were also presented in the three afore-mentioned conditions despite the fact that the volunteers had not to discriminate between their types and gave an overt motor response using alternatively only the index fingers of the two hands depending on the target arrowhead orientation.

The ANT-SR [40] was used to investigate the effects of hypoxia on endogenous and exogenous visuospatial attention orienting modes. Capitalizing on the different set of alerting and attention-orienting cue-target conditions of the redesigned ANT-SR test [40,44], and assuming that an increase of alpha synchronization represented an inhibition of attention orienting, we hypothesized that:

1. an increase in alpha power would have been found for the spatially uninformative CC condition as compared to both the LC and NC conditions;
2. the addition of a perceptual and motor-choice workload (as in the LCmot condition) would have adversely affected overt motor responses and brain attention orienting capacity as reflected by an increase of alpha oscillations;
3. alpha power would have been affected differently by exogenous and endogenous attention orienting modes over the two hemispheres;
4. hypoxia would have resulted in a general increase in alpha power in both exogenous and endogenous attention orienting modes.

## 2. Materials and Methods

### 2.1. Study Compliance with Ethical Standards and Participants

The study was approved by the ethics committee of the Italian National Research Council (CNR) and was conducted in the Electro-Functional Brain Imaging unit (EFBIu) of the CNR-IBFM Institute in accordance with American Psychological Association (APA) ethical standards for the treatment of human experimental participants (APA, Monitor Staff, 2003, vol. 34, n. 1). Furthermore, the experiments were conducted with the understanding and the written consent of each participant in compliance with the indications of the 2018 Declaration of Helsinki ethical principles for medical research involving human subjects by the World Medical Association (WMA Declaration of Helsinki, 9 July, 2018, PDF file).

Ten (10) healthy volunteers (4 females and 6 males), with age ranging from 19 to 27 years (Mean = 24, SD = 2.7) were selected for participating in randomized order in two EEG recording sessions during which they breathed either ambient air (also indicated as “normoxia”) or a 12.5% O_2_-impoverished air mixture (also indicated as “hypoxia”). Besides suffering or having suffered of any neurological and psychological syndromes or the intake of any psychopharmacological substances, criteria of exclusion from the study were cigarettes smoking, arterial hypertension, and cardiovascular or respiratory diseases. In addition, to minimize confounding effects, no participant had to have sojourned at a higher altitude than 300–400 m in the 4 weeks preceding the study nor had to have been regularly and intensively engaged in any physical training program. Again, volunteers were required to refrain from any strenuous physical activity and from unlimited consumption of alcohol, caffeine, and theophylline containing beverages in the 24 h prior to the experimental sessions of the study. All selected participants had normal or corrected–to–normal vision and right-eye as well as right-hand dominance and none of them had any left-handed relatives as assessed by the Edinburgh Inventory. Unfortunately, the data of two volunteers had to be excluded from the statistical analyses either because of excessive eye—and body—movement artifacts during EEG recordings or for not completing the two recording sessions cycle.

#### Sample Size Limit

The small size of the sample here used introduced a risk of underpowered statistical computations in the study. However, we kept this risk under thorough control estimating the effect size for the statistically significant factors by means of partial eta squared values, i.e., $\eta_{p}^{2}$. Additionally, the alpha inflation due to multiple comparisons was also controlled by means of Greenhouse–Geisser epsilon (ε) correction.

### 2.2. Stimulus Materials and Experimental Conditions

**Figure 1:**
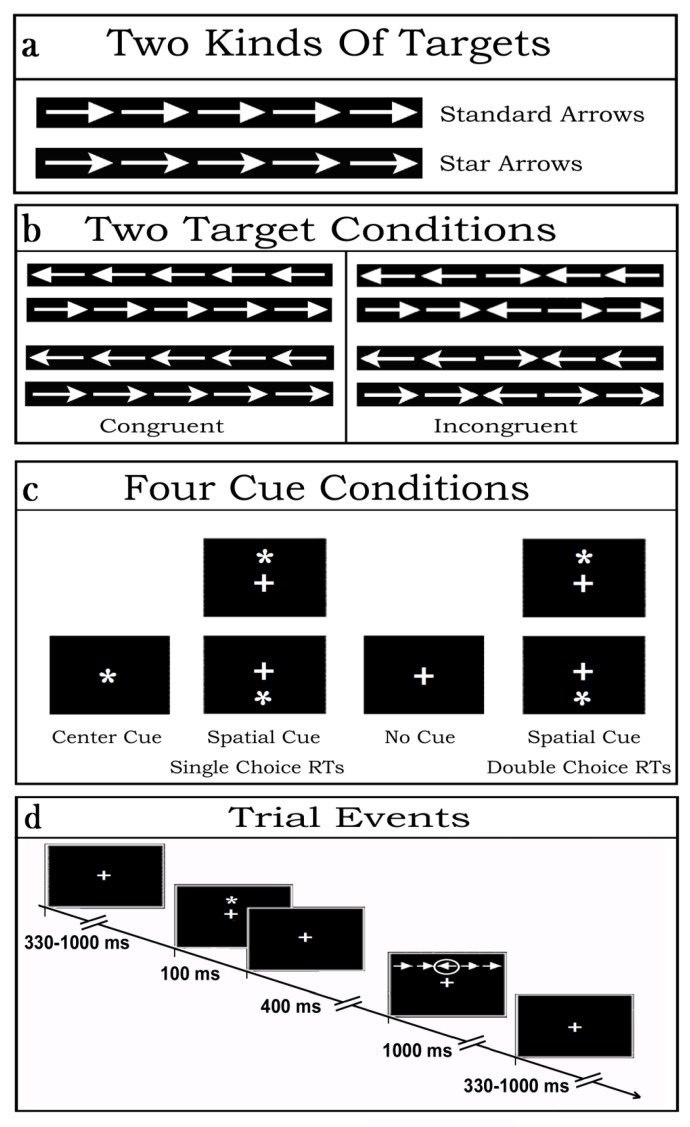
Graphical depiction of the stimulus materials, of cue-target conditions, and of trial events used in the present study (ANT-SR (Attention Network Test-Slightly Redesigned); modified and redrawn from Fan et al. [40] and Posner [44]). (**a**) Two kinds of white arrow-target strings were presented: “standard” and “star” arrows. (**b**) Congruent and incongruent central targets vs. flanker strings were presented. Based on the direction of the arrow tip (left or right), eight target-flanker conditions resulted. (**c**) Four different cue-target and motor-task experimental conditions were administered in randomized order: a no cue (NC) condition; a center, alerting but spatially unpredictable cue (Central Cue, CC), and a valid spatially informative cue (Local Cue, LC). In these three conditions, participants’ motor response to targets depended on a single-choice reaction time (RT) button-press. In a fourth cueing condition, LCmot, volunteers had to discriminate both the target-arrow type and orientation in order to make a double-choice RTs button-press. (**d**) Schematic exemplification of cue–target stimulus-events and inter-stimulus interval (ISI) duration during a single trial of the LC condition as well as of the random inter-trial interval (ITI) length. See text for further details.

Stimulus materials consisted of strings of five (5) contiguous arrows, serving as targets. Arrows were of two types: so-called “standard” and “star” arrows. While the former had a tip with a vertically linear rear side, the latter showed a slightly inward-bound and oblique rear-side at the starting of their shaft (see Figure 1a). The central arrow of each string consisted of the true target while the flanking two arrows on each side of the latter posed as potential distracters. Overall, each target-and-flankers-string subtended 8.7 degrees of visual angle along the horizontal meridian and 1.3 degrees along the vertical meridian. Regardless of the arrow-type, the tip of the central target arrow could point to the left or to the right side, whereas the flanking arrows could point toward the same (Congruent flankers) or the opposite direction (Incongruent flankers) as the target. All in all, then, there were eight (8) different target strings combinations (see Figure 1b for examples of the latter).

Prior the presentation of arrow-strings, stimulation also included the administration or the omission of white asterisks in different points in space of the stimulation PC monitor so to produce four different cue-target conditions. All these stimulus materials were presented on the blackened background of a 17“- cathode-ray tube (CRT) screen in front of the volunteers. The luminance of both the asterisk-cue and the two types of target-arrow-strings were measured in candela/m^2^. The luminance of the asterisk amounted to 7.3 candela/m^2^. Conversely, the luminance assessments for the standard-arrow- and star-arrow-strings were 27.81 and 26.96 candela/m^2^, respectively, and were matched across arrow-pointing directions and target-flankers congruency.

Depending on the cue presentation positions (above or below the fixation cross (FC) or centered over it) or omission, as well as to target-related motor tasks to be performed, four (4) different attention alerting or valid spatial attention orienting sets were induced in the participants (Figure 1c). More in details, the latter had to deal with: (1) the sequential presentation at random of vertically eccentric (above or below the FC) target strings without being preceded by any cue aimed at eliciting a possibly tonic and unspecific alerting or sustained attention condition over time (No Cue, NC) as well as a target-related exogenous spatial orienting of attention to the point in space where the arrow-strings were contingently presented from trial-to-trial; (2) the presentation of a cue overimposed on the FC aimed at eliciting a phasic attention alerting but not an attention orienting response followed by the presentation of a target string above or below the FC so to elicit a target-related exogenous spatial orienting of attention to the point in space where the target-string was delivered (Central cue, CC); (3) the presentation of a vertically eccentric (above or below the FC) cue aimed at eliciting both a cue-related phasic attention alerting and an exogenous orienting of attention to the point in space indicated by the cue, later on followed by a further endogenous orienting onto the target at that same point because the focus of attention was already centered there (Local or Spatial cue condition, LC).

For each of these three cueing conditions of the original ANT, on each trial the participants had to discriminate the direction towards which the central target-arrow-tip of the five-arrows-string pointed, independent of the arrow type presented (i.e., standard or star arrow), and of the direction of the flanking-arrows (Congruent and Incongruent), and to perform a single-choice-RTs button-press with the index finger of the corresponding hand (right or left). Participants had also to deal with a fourth, newly introduced, cueing condition of the ANT-SR, defined LCmot. The latter was like LC but demanded a double-choice-RTs button-press according to the target-arrow type presented. Indeed, during the LCmot condition participants had to discriminate the type of target-arrows (i.e., standard- or star-target-arrow), besides the direction to which the latter were pointing to, in order to perform a double-choice button-press with the index or middle fingers, respectively, of the corresponding hand (right or left, respectively; See Figure 1b,c again).

No matter the cue-target condition considered, on each trial the cue was presented for 100 ms followed after 400 ms by the delivery of an arrows-string, which remained on the screen for 1000 ms before its offset in order to avoid any possibly baffling stimulus-offset, besides stimulus-onset, related ERP recordings. Inter-trial interval (ITI) randomly varied between 330 and 1000 ms (Figure 1d).

### 2.3. Procedure

The selected participants took part in randomized order in two 4 h lasting EEG recording sessions, one week apart from each other. During these two experimental sessions they breathed either ambient air (or normoxia) or a 12.5% O_2_-impoverished air mixture (simulating respiration conditions at an high altitude of ~4200 m (~13,780 feet) at sea level), which may be assimilated to an acute bout of tolerable and far from pathogenic normobaric hypoxia while performing in four different cue-target visuo-spatial attention conditions taken and readapted from Fan’s et al. [40] and Posner’s [44] ANT.

**Figure 2:**
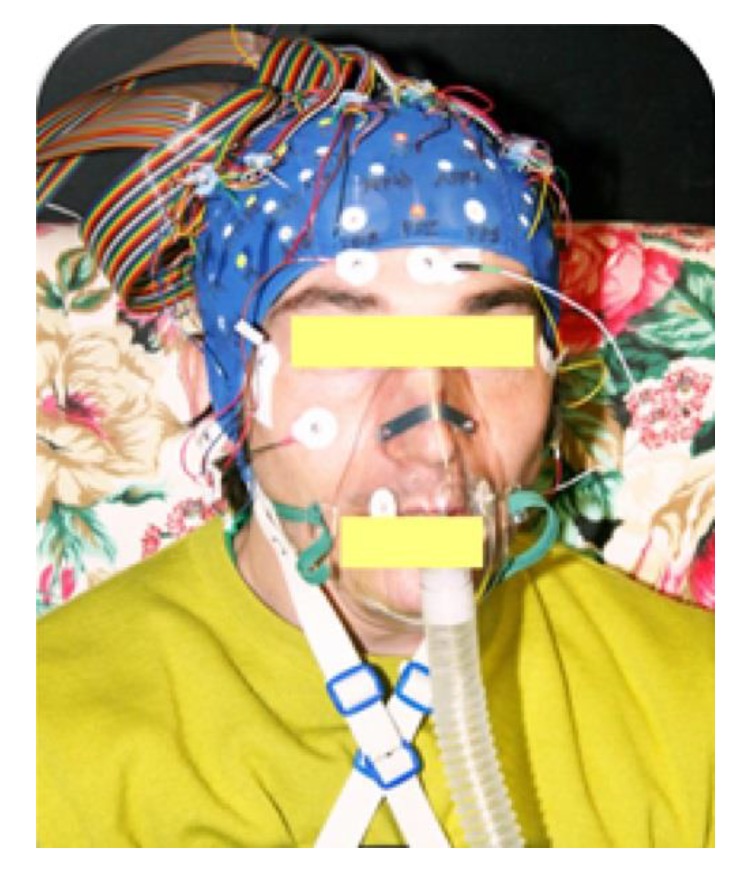
A picture of a participant wearing the mask connected to the hypoxicator apparatus which provided air impoverished of a 12.5% of oxygen during the electroencephalogram (EEG) recording session.

Independent of the respiratory session with which the volunteers started their participation in the study, while being prepared for the EEG recording during their first report to the lab, they underwent a general psychophysiological screening that included the chronicling of their chronobiological daily habitual activity patterns (Morningness Eveningness Questionnaire—MEQ [45]). In addition, we also measured their subjective mood and alert levels by means of so-called paper-and-pencil psychophysiological Likert-like scales and three minutes speeded-up letter-cancellation tasks. As for the hypoxia session, just starting from their arrival to the laboratory the volunteers breathed a normobaric hypoxic mixture obtained removing a controlled amount of oxygen from air (i.e., 12.5% O_2_) by means of a MAG-10 hypoxicator apparatus (Higher Peak LLC, Winchester, MA, USA). The mixture was delivered through a facemask at 30 L/min. Excess air flow was diverted outside the mask to prevent inspired oxygen pressure from increasing above 90 Torr (see Figure 2 for a visual exemplification).

Since there are influential data in the physiological literature indicating that in humans the most relevant cardiorespiratory and blood plasmatic effects were found in between 2–4 h of serious hypoxic respiration (see, e.g., [46]), all our volunteers were systematically submitted to the EEG recording session starting after 2 h of such a respiratory condition, which was spent in the application and jellying of a 128 electrodes electrocap as well as in the filling of the mood and alert paper-and-pencil scales, besides in an alert letter-cancellation-based task. For experimental standardization and session comparison-sake, the volunteers started their tasks-related EEG recordings after 2 h during the normoxic session too, a span of time overall spent in the activities mentioned above besides the electrocap application.

At the end of the preparation time-span, participants were invited to take a seat in a comfortable easy chair with a high backrest within an electrically and magnetically shielded cubicle (Faraday cage) in front of a CRT screen with a small white fixation cross (FC) in the center of its black background placed at a distance of 114 cm (or 3.34 feet) from them. During the EEG recordings, sequences of the white arrow-strings were randomly presented above or below the fixation-cross at the center of the visual screen. The central target-arrow of each string fell just in correspondence of the fixation cross with a vertical eccentricity of ±1.25 degrees of visual angle from the latter. Apart for the NC condition, an asterisk-cue always preceded the arrow-strings in the CC, LC, and LC mot conditions.

To familiarize participants with the task to be performed in each cue-target condition, before recording was started volunteers read detailed written instructions and performed a practice run of 30 stimulus pairs. For each of the four cue-target condition, there were four separate blocks of trials, each containing 128 trials grouped in a differently randomized order and lasting approximately 3.5 min. To avoid any confounding and systematic interacting effects of practice, fatigue and hypoxia, we also randomized the order of administration of the respiratory conditions, of the cue-target conditions, of the type of target-arrows, of the falling of the target in the upper or lower visual field, and of blocks presentation across participants.

### 2.4. EEG Recording and Analysis

For EEG recordings we used scalp electrodes mounted in an ANT elastic Waveguard 128-electrodes electrocap. The electrodes were densely spaced all over the frontal, central, temporal, parietal, and occipital scalp-sites as proposed by the 5% system [47] devised for high-spatial resolution EEG/ERP recordings. Two electrodes placed below and above the right eye recorded vertical eye movements, whereas two further electrodes placed at the outer canthi of the eyes recorded horizontal eye movements. Linked ears served as the reference lead, whereas a frontal electrode served as a ground site. Electrode impedance was below 5 kΩ. Both EEG and electrooculogram (EOG) continuous signals were acquired using directional-current (DC) amplifiers and a digitization rate of 512 samples/s. Offline, automated rejection of electrical artifacts was performed before EEG averaging to discard epochs in which eye movements, blinks, or excessive body muscle potentials occurred. The artifact rejection criterion was a peak–to–peak amplitude exceeding ± 70 μV for EEG signal or ± 100 μV for EOG signal, and, in line with the trials stimulus events timing, went from 100 ms before the cue-type to 1500 ms after it for each trial in sequence, until the routine detected EEG values falling within the indicated window-values. Overall, the rejection rate was ~6.0 %. Although we used the indicated rejection method implemented in our EEG signals analysis applications package, we are aware that other independent component analyses would have quite efficiently worked [48,49]. Trials associated with missing (i.e., overt motor responses falling after 1000 ms from the target) were also discarded, which resulted in a rejection rate of less the 2%. Overall, then, a global 92% out of the 100% trials administered were accepted for averaging. Trials associated with motor-response errors (i.e., on the one hand, responses given with the wrong hand with respect to the left/right target-arrow pointing directions, no matter the arrow types, for the NC, CC, and LCmot conditions, and, on the other hand, the wrong finger with respect to the standard/star arrow types, besides with the wrong hand, for LCmot) were also computed separately as a function of respiratory conditions and discarded from EEG averaging. So, after rejection procedures and the due mean computations over the sample of participants, the mean percentages of correct trials averaged as a function of NC, CC, LC, and LCmot conditions in normoxia amounted to 98%, 99%, 99%, and 94% of the 100% of total trials administered, respectively, and to 98%, 95%, 94%, and 89%, respectively, in hypoxia. More specifically, in normoxia the percentages of congruent vs. incongruent trials averaged for the former congruency level of NC, CC, LC, and LCmot conditions were 50.41%, 49.79%, 50.69%, and 47.29% of the 100% total trials administered, respectively, and 48.35%, 48.83%, 49.15%, and 46.65%, respectively, for the latter congruency level. In hypoxia the percentages of congruent vs. incongruent trials averaged for the congruent target trials of the four cueing conditions were 49.90%, 48.61% 47.50%, and 45.54% of the 100% total trials administered, respectively, and 48.32%, 47.23%, 46.70%, and 43.42%, respectively, for the incongruent target trials.

Before selective averaging, accepted EEG signal chunks were digitally filtered with a half–amplitude band-pass of 0.016–70 Hz. As for the rejected epochs, accepted EEG epochs in the averages were synchronized with the onset of cue presentation (CC, LC, and LCmot conditions) or omission (NC condition) and went from 100 ms before the cue-type to 1500 ms after it, the target-related EEG being elicited 500 ms after cue-onset. In order to compare the averaged EEG epochs relative to the single-choice motor-workload conditions (i.e., NC, CC, and LC) with those generated in the double-choice motor-workload condition (LCmot), EEG responses to the different types of arrow-targets (i.e., standard and star arrows) were collapsed together in all cueing conditions. Furthermore, in order to increase the signal-to-noise ratio EEG trials related to targets delivered in the upper and lower visuospatial hemifields were also collapsed together in all cueing conditions.

Hence, for each subject distinct EEG average waveforms were obtained according to respiratory condition (i.e., ambient-air or hypoxia), cueing-task condition (CC, LC, NC, LCmot), and target-congruency (i.e., congruent, incongruent) condition. Besides average EEG waveforms for each single participant, grand-average EEG signals were also computed for the participants’ sample.

### 2.5. Alpha Power Analysis

To obtain the alpha power as a function of the different experimental conditions, the Fast Fourier Transform (FFT; [50]) computation was used. The reference point for the FFT analysis or Start Time was the time of cue presentation, at 0 ms latency, while the End Time was the 1500 ms latency. The time span of the EEG waveforms was divided in three (i.e., 3) blocks, each with a length of 500 ms, with a sample count which had the power of two (i.e., 512 Hz). Alpha oscillations content and, more specifically, alpha amplitude in the range of 7.5–12.5 Hz was computed and averaged over the three blocks. To allow a better comparison of FFT results over experimental conditions, a normalization of the computed alpha power in the aforementioned band with respect to the summed power over all frequency values of the channels with the highest power was carried out, and, as a whole, reported in µV^2^. Additionally, the baselines of the spectral analyses were corrected across conditions before FFT computation. These procedures were automatically applied to each site of the electrodes montage so to obtain a topographic distribution of the alpha power spectra over the scalp as a function of the experimental variables. Topographical voltage maps of alpha were obtained by plotting color-coded alpha power values derived by interpolating frequency content values between scalp electrodes as a function of respiratory and cueing conditions. Eye-balls inspection of the maps indicated that EEG alpha power reached higher amplitude values at posterior than anterior scalp regions regardless of respiratory and attention cueing conditions, and that the topographic distribution of alpha amplitude at posterior scalp regions changed as a function of experimental conditions considered. In order to test the statistical significance of these apparent data changes, taking into account previous EEG findings in the literature, alpha power amplitude was measured at four posteriorly-anteriorly distributed couples of homologous electrode sites: O_1_ and O_2_, mesial-occipital electrodes; PO_7_ and PO_8_, lateral parietal-occipital electrodes; TPP_7h_ and TPP_8h_, temporo-parietal-parietal electrodes; and F_5_ and F_6_, pre-frontal lateralized electrodes.

## 3. Statistical Analyses

### 3.1. Statistical Analyses for Behavioral Data

Behavioral data, namely both motor response errors and speed (reaction times—RTs), underwent two separate 3-way repeated-measures ANOVAs whose factors of variability were: Respiratory conditions (2 levels: Air and Hypoxia), Cueing conditions (4 levels; NC, CC, LC and LCmot) and Target congruency (2 levels: Congruent, Incongruent). Before being submitted to the multifactorial repeated-measures ANOVA, error rate percentages were converted to arcsine values because percentage values do not exhibit homoscedasticity (e.g., [51], which is necessary for ANOVA. In fact, the distribution of percentages is binomial, whereas the arcsine transformation of the data makes the distribution normal [52].

### 3.2. Statistical Analyses for Electrophysiological Data

Alpha power measures were submitted to a five-way repeated–measures ANOVA with respiratory condition (R, 2 levels: Ambient-air and Hypoxia), attention cueing condition (AC, 4 levels: NC, CC, LC, and LCmot), arrows-target array congruency (C, 2 levels: Congruent and Incongruent), hemisphere (H, two levels: left hemisphere, LH, and right hemisphere, RH), and electrode (E, 4 levels; O_1_–O_2_, PO_7_–PO_8_, TPP_7h_–TPP_8h_, and F_5_–F_6_ electrode sites).

For both behavioral and electrophysiological data, the partial eta squared values ($\eta_{p}^{2}$) were systematically provided to estimate effect sizes [53,54]. Additionally, (ε) Greenhouse-Geisser correction was applied to compensate for possible violations of the sphericity assumption associated with factors which had more than two levels. The epsilon (ε) values and the corrected probability levels (in case of epsilon < 1) are reported. Post-hoc comparisons among means for significant factors with more than two levels were performed by means of Tukey HSD and/or Newman–Keuls tests.

## 4. Results

### 4.1. Behavioral Results

#### 4.1.1. Error-Percentage Rates

**Figure 3:**
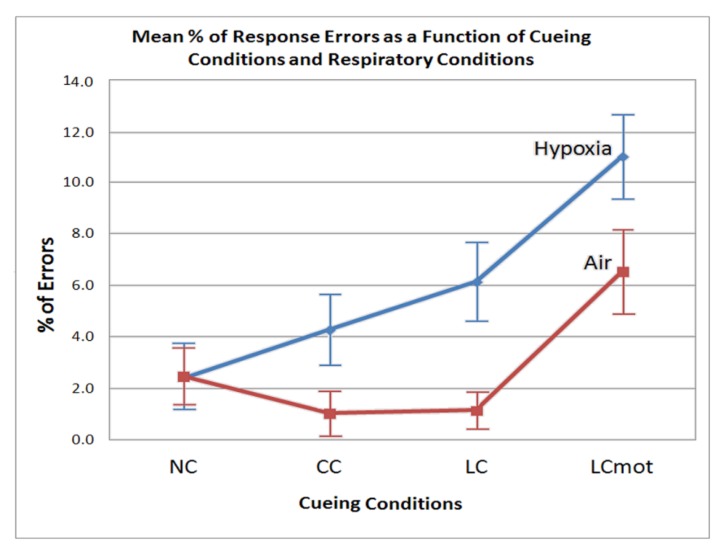
Mean percentages (along with standard errors (SE)) of errors committed by participants as a function of cueing condition and respiratory condition regardless of the target strings congruency.

The ANOVA carried out on motor response errors proved the significance of the main “respiratory condition” factor (F (1,7) = 7.276: *p* < 0.025)), indicating that in air participants showed a 2.51% of errors, while during hypoxia this rate increased on average to 6.12%. In addition, error percentage-rates for the various cueing conditions significantly changed as a function of the respiratory condition (F (2.63, 18.45) = 31.25; ε = 0.879; adjusted *p* value < 0.000024; $\eta_{p}^{2}$ = 0.61). Post-hoc comparisons indicated that, in air, error rates for NC were higher than for both CC (*p* < 0.0001) and LC (*p* < 0.0001), but lower than for LCmot (*p* < 0.0002). In turn, CC did not differ from LC, but it showed a lower errors rate than LCmot (*p* < 0.0001). Again, LC obtained a lower errors rate than LCmot (*p* < 0.0001; see Figure 3 for these findings.)

In hypoxia, instead, the post-hoc contrasts indicated that error percentage rates progressively and significantly increased from NC to LCmot cueing conditions: NC vs. CC, *p* < 0.0002; NC vs. LC, *p* < 0.0001; NC vs. LCmot, *p* < 0.0001, respectively. Moreover, CC was also different from LC (*p* < 0.0002) and from LCmot (*p* < 0.00001), and, in turn, LC was different from LCmot (*p* < 0.0001). Due to the aforementioned increases, with the exception of NC, the CC, LC and LCmot cueing conditions showed significantly higher error rates in hypoxia than in air (i.e., *p* < 0.001 for CC; *p* < 0.0001 for LC; *p* < 0.000025 for LCmot; see Figure 3 again).

#### 4.1.2. Reaction Times (RTs)

The ANOVA carried out on mean RTs showed that the measures obtained for the two levels of the respiratory factor (i.e., Air: Mean = 458.60 ms, standard error (SE) = 13.68; Hypoxia: Mean = 474.21 ms, SE = 15.01) were significantly different (F (1,7) = 4.799; ε = 1; *p* < 0.05, $\eta_{p}^{2}=$ 0.51). The ANOVA also yielded significant effects of cue-type (F (2, 16; 15, 15) = 176.96; ε = 0.72; adjusted *p* value < 0.00001, $\eta_{p}^{2}=$ 0.96) and target flanker-type (F (1, 7) = 167.23; *p* < 0.000004) and a significant interaction between the two (F (2,58; 18,08) = 39.96; ε = 0.86; adjusted *p* value < 0.000002, $\eta_{p}^{2}=$ 0.85).

**Figure 4:**
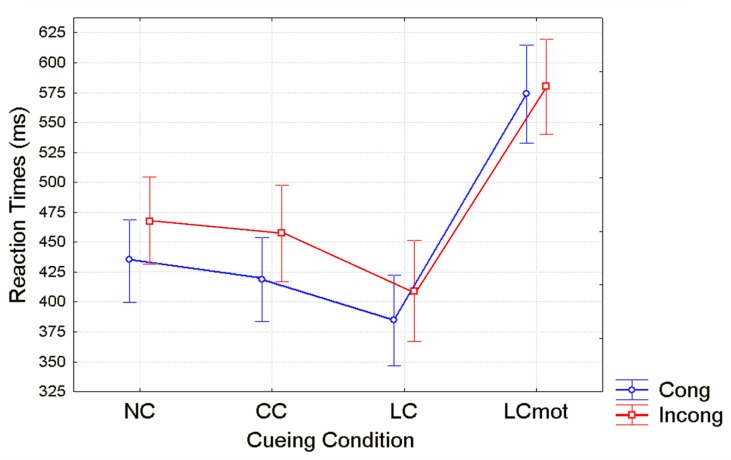
Mean RT and Standard Error (SE) values obtained as a function of the cueing condition and target congruency over the sample of participants. Note that RTs were measured starting from the target delivery time, that is 500 ms later than the cue omission or administration according to the cueing condition.

Post-hoc comparisons showed that, except for the LCmot cueing type, response times were much faster for all the other cueing conditions when target-strings were congruent than incongruent (i.e., NC (*p* < 0.00002), CC (*p* < 0.0002) and LC (*p* < 0.0002), respectively; see Figure 4). Post-hoc contrasts also indicated that RTs were slower for NC than for both CC (Cong: *p* < 0.0002; Incong: *p* < 0.0002) and LC (Cong: *p* < 0.0002; Incong: *p* < 0.0002), and in turn for CC than LC for both target flanker types (Cong: *p* < 0.0002; Incong: *p* < 0.0002). Further contrast analyses proved that LCmot obtained much slower RTs than the other three cueing conditions, namely NC, CC, and LC, for both target congruency conditions (Cong: LCmot vs. NC, *p* < 0.0002; LCmot vs. CC, *p* < 0.0002; LCmot vs LC, *p* < 0.0002; Incong: LCmot vs. NC, *p* < 0002; LCmot vs. CC, *p* < 0.0002; LCmot vs. LC, *p* < 0.0002; see Figure 4 again).

### 4.2. Electrophysiological Results

**Figure 5:**
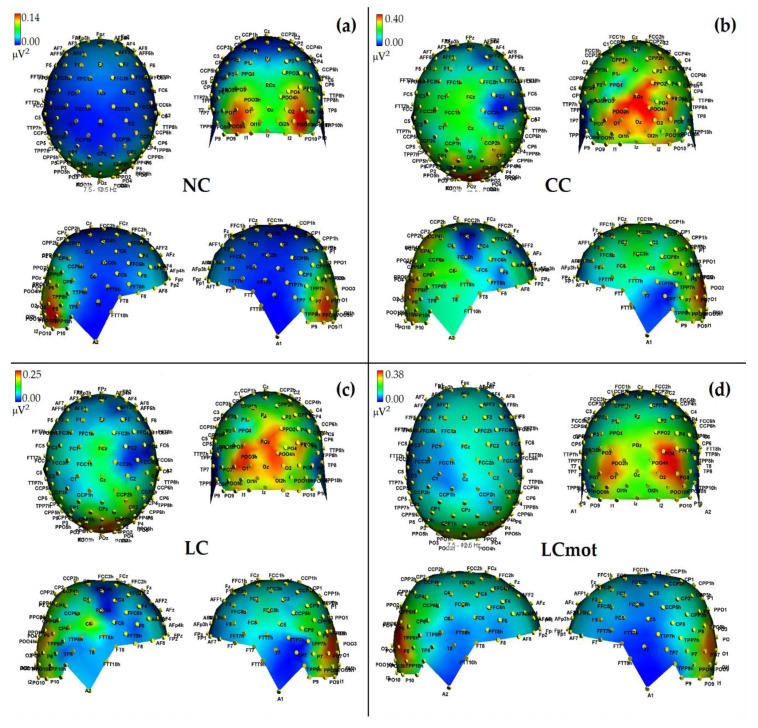
Three-dimensional (3D) maps of the topographical distribution of alpha power over the scalp as a function of the cueing-conditions grand-averaged over the respiratory conditions, and drawn from the top, the back, the right, and the left points of view, respectively. Time = 0 to 1500 ms, frequency = 7.5–12.5 Hz. (**a**): NC condition; (**b**): CC condition; (**c**): LC condition; (**d**): LCmot condition. Note that the scale of alpha power values (in µV^2^) is unalike in the different attentional-cueing conditions. Note also that while the alerting but spatially unpredictable, exogenous-orienting of attention to target (CC) condition showed the highest alpha power, the fully uncued, exogenous-orienting of attention to target (NC) condition was characterized by the lowest alpha power.

**Figure 6:**
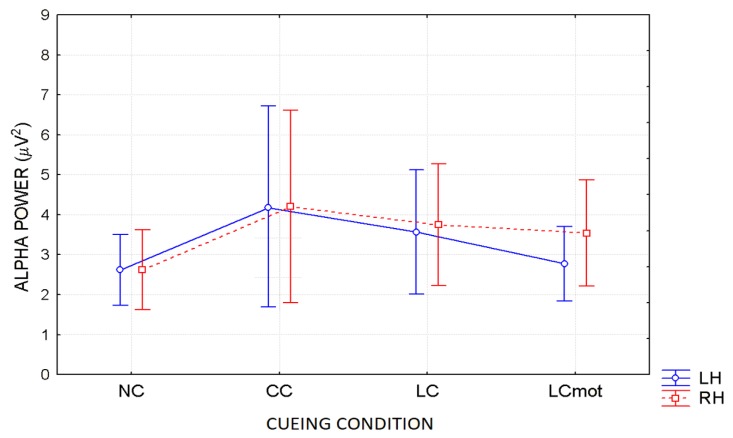
Mean and SE values of alpha power (in µV^2^) as a function of cueing conditions and hemispheres collapsed across respiratory conditions. Note that for an easier readability, alpha power values were multiplied by 10 before being plotted.

ANOVA showed the significant interaction of Cueing condition x Hemisphere (F(2.17, 15.19) = 6.16; ε = 0.72; adjusted *p* value < 0.00001). Effect size for this significant interaction was $\eta_{p}^{2}$ = 0.47. Post-hoc comparisons among means showed that alpha power was significantly stronger over the RH than LH for the LCmot condition only (*p* < 0.000028; for these effects see maps of Figure 5a–d as well as the mean and SE values drawn in Figure 6).

Further post-hoc contrasts between the alpha power values obtained for the different cueing conditions within the two hemispheres also indicated that alpha power was overall greater in the CC than NC condition over both hemispheres (i.e., *p* < 0.00001 for LH, and *p* < 00001 for RH, respectively). Alpha power was also greater in the LC than in the NC condition over both hemispheres. Conversely, alpha power was decreased in the LCmot compared to the CC condition (*p* < 0.00001 for LH, and *p* < 0.0033 for RH, respectively). Post-hoc tests also showed a difference between alpha power values for both the spatially informative conditions LC and LCmot over the LH (*p* < 0.00001), but not the RH. Again, alpha showed an enhanced power in the CC than in the LC condition over the LH (*p* < 0.008), but not the RH, despite an intriguing trend (*p* < 0.062).

**Figure 7:**
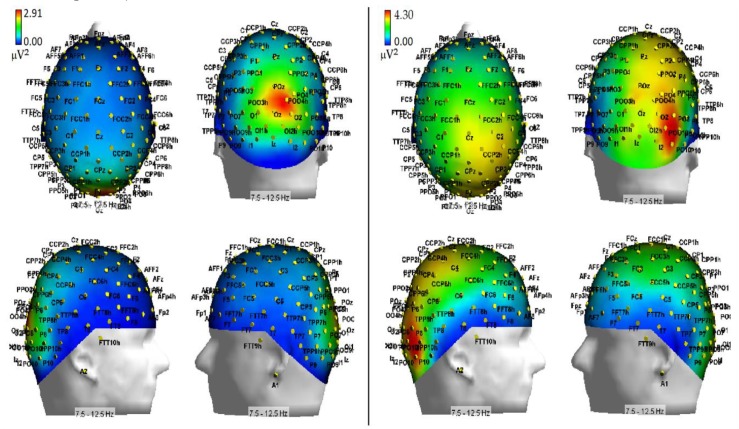
3D topographical scalp maps of alpha power as a function of normoxia (left side) and hypoxia (right side) respiratory conditions grand-averaged across the attentional-cueing modes, and drawn from the top, the back, the right, and the left viewpoints, respectively. Worth of note is the prominent lateralization of alpha synchronization induced by hypoxia over the right-sided occipital and parietal scalp areas as compared to normoxia.

**Figure 8:**
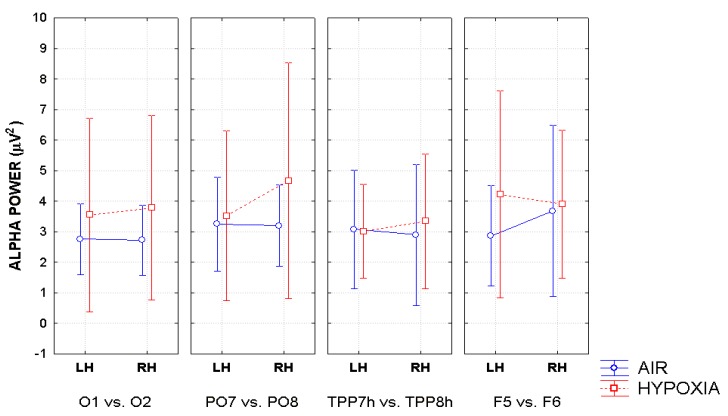
Mean and SE values of alpha power plotted as a function of respiratory mode, hemisphere, and electrode site. Note that also in this case alpha power values were multiplied by 10 before being plotted.

The ANOVA also yielded a significant triple interaction of Respiratory condition, Hemisphere and Electrode factors (F (1.74; 12.15) = 3.72; ε = 0.58; adjusted *p* value < 0.05; effect size $\eta_{p}^{2}$ = 0.35) regardless of the cueing condition (For the topographic distribution across electrode sites and hemispheres in the two respiratory conditions see the maps drawn in Figure 7), while for the mean and SE values relative to the afore-mentioned findings see Figure 8). Post-hoc comparisons among means indicated that hypoxia significantly increased alpha power (*p* < 0.0005) determining a prominent alpha synchronization over the right-sided parieto-occipital scalp site (PO8), but not the left occipito-parietal (PO7) site. Furthermore, alpha power was greater over the right (PO8; *p* < 0.004) than the left occipito-parietal (PO7) site.

Additionally, hypoxia induced EEG alpha synchronization over the mesial-occipital areas of both the LH and RH (*p* < 0.04 and *p* < 0.0072, respectively). Hypoxia also induced stronger alpha synchronization than in ambient-air over the left-sided dorsolateral prefrontal scalp site (*p* < 0.0011) (see Figure 8 again for all these findings). Overall, however, at these anterior scalp regions EEG alpha synchronization was significantly stronger over the RH than the LH, but in air condition only (*p* < 0.034). Moreover, in hypoxia alpha synchronization showed to be much more prominent over the right parieto-occipital than over the temporo-parietal (*p* < 0.001) and the prefrontal (*p* < 0.046) scalp sites, while this topographical difference was much reduced in ambient-air condition and over the left hemisphere (*p* < 0.0011).

## 5. Discussion

The present study had the manifold aims of investigating the role of alpha synchronization and/or desynchronization in visuospatial attention orienting, and, more specifically, in the exogenous and endogenous modes of attention-orienting. Additionally, we aimed to investigate the relationships of alpha synchronization and desynchronization with the functional activation of brain alerting, orienting, and executive attentional neural networks. We wished to assess whether the separate attentional networks might have been independently or interactively affected by a reduced, non-pathogenic brain oxygenation state—or hypoxia—and, in case of interactions, which of them would have interacted. Last but not the least, we inquired into possible influences of induced hypoxia on overt motor performance.

From the behavioral point of view, our findings indicated that both error rates and RTs showed to be affected by hypoxia, in that, during this respiratory condition, the participants showed a tout court lower performance accuracy and speed than in air—or normoxia—regardless of cueing mode and target/flanker congruency.

However, error rate differences between cueing conditions also showed to be strongly affected by the respiratory factor regardless of target/flanker congruency. In normoxia, in fact, the lowest error rate was observed for both the spatially cued (LC) and the alerting but spatially unpredictable, centrally cued (CC) targets, without any difference between them (see Figure 3). A higher errors rate was obtained for uncued targets (NC), and a highest rate for spatially cued, motor-choice overloaded (LCmot) targets. Conversely, with the exception of uncued targets (NC), hypoxia markedly affected performance accuracy in that the latter progressively decreased—i.e., participants committed a greater number of errors—as a function of both the alerting but spatially unpredictable, centrally cued (CC) and spatially cued targets, regardless of their demand of a single or a double-choice motor response (i.e., LC and LCmot). All in all, these overt motor-accuracy findings suggested that hypoxia affected the original ANT-related attention alerting, orienting, and conflict networks independently from one another. Moreover, our data suggested that hypoxia also strongly affected the motor-charged attention orienting cueing mode.

Unlike for error rates, overt responses (i.e., RTs) showed a distinct pattern of significant changes as a function of cueing conditions in close interaction with target/flanker conflict level, regardless of the respiratory condition. Shortest RTs were observed for validly spatially cued (LC) targets, intermediate RTs for the spatially unpredictable, centrally cued (CC) targets, longer RTs for uncued targets (NC) and longest RTs for spatially cued, motor double-choice demanding (LCmot) targets (see Figure 4). The relative benefits of original ANT alerting and orienting [40] amounted to about 25 ms and 50 ms, respectively, while for the motor double-choice-related orienting condition of our ANT-SR, a heavy cost of about 200 ms, rather than a benefit, was found. Response conflict due to target/flankers incongruency led to an increase of overt motor response latency with a greater mean RTs cost of about ~40 ms for the spatially unpredictable, centrally cued (CC) targets than for both the uncued (NC; ~30 ms) and the validly spatially cued targets (LC; ~20ms), and no cost for LCmot cueing condition.

At least for what concerned the behavioral findings in response to the original ANT-related cueing conditions in normoxia, the aforementioned pattern of results is in good accordance with those that were published originally by Fan et al. [40], Neuhaus et al. [55], and by our group (e.g., Zani and Proverbio [43]), in that the use of either a spatially informative cue (LC) or of a no-cueing condition (NC) reduced costs for incongruent vs. congruent targets processing as compared to both the 2C and CC cueing modes. According to Fan et al. [40], this occurred, in the first case, because participants directed attention to the target ahead of time, and, in the second case, because NC is a relatively low-alertness condition and resulted in longer RTs and relatively lower errors. In Fan’s et al. [40] words, “it is possible that the longer time to produce a response due to low alertness can provide additional time for executive attention processes in the conflict condition, thus reducing executive costs.” Following the above-mentioned lines of reasoning, it would seem highly plausible that these same neural mechanisms could be adopted for explaining the lack of any differences between the speed of motor response to the congruent and incongruent target/flanker patterns for the motor-charged, LCmot condition (see Figure 4 again).

Generally speaking, we believe that the consistency of our behavioral findings with those obtained by the studies quoted above strongly supports the views that, despite the relatively exiguous number of participants analyzed in the present study, the obtained data may be considered sound and reliable, besides being truly dependent on the manipulation of experimental variables.

As far as the electrophysiological data are concerned, our findings showed to be quite in accordance with those obtained for behavioral data. Indeed, alpha measures separately changed as a function of cueing condition and respiratory condition factors, either in interaction with the hemisphere and/or the hemisphere and electrode factors. Unlike behavioral data, however, electrophysiological data were not apparently affected by the executive neural processing, neither per se or in interaction with other factors.

It would seem rather plausible that this slight inconsistency between overt motor responses and covert alpha processing data depended on the fact that the former were timely closer to the executive conflict resolution processes than the latter because alpha power computed by FFT starting from the presentation of cue type, at time 0, up until 1500 ms after cue, with the target falling at 500 ms, included, according to the experimental conditions, time spans of either alpha synchrony or desynchrony occurring after the emission of the overt motor response. Due to this, it is somehow conceivable that alpha power processing occurring during these time spans may have been less closely related to the executive conflict resolution processes.

As for the specific trend of our electrophysiological data, it is worth of note that, notwithstanding the small size of our sample of participants, our findings are in line with those of previous studies, thus confirming the view that a bout of increased alpha amplitude or synchronization recorded at posterior occipito-parietal areas during a visuospatial attention task reflects an inhibition of attention orienting towards information presented at an irrelevant point in space [56]. Indeed, brain areas involved in the processing of task-irrelevant space may be actively inhibited during an increase of alpha-oscillations synchronization to refrain from processing of distracting information [19,57]. More in details, the data showed that alpha power changed not only as a function of endogenous and exogenous attention orienting modes, but also of the different informative content transmitted by the various cue-target combinations within the former and the latter modes of attention-orienting, regardless of brain oxygenation condition. They also indicated that attention-related alpha frequency desynchronization is more prominent in the LH than in the RH, thus suggesting asymmetries that might be regarded as a sign of cerebral lateralization or hemispheric dominance for this neurocognitive function.

In agreement with these viewpoints, alpha showed the lowest power (i.e., stronger desynchronization) during the no cue (NC) mode, in which, most likely, brain tonic alerting and utterly exogenous orienting of attention responses to targets sequentially presented in random order at one out of the two relevant space locations without being primed by any cue, took place (see EEG alpha-power maps drawn in Figure 5a, and Figure 6 for mean power values for this condition). At the opposite, alpha showed the highest synchronization in the ANT condition in which a spatially uninformative, but alerting cue was delivered to the center of the screen, followed later on by the presentation of a target at one out of the two spatially relevant locations (i.e., CC condition; see EEG alpha-power maps drawn in Figure 5b, and Figure 6 for mean power values for this condition). Very likely, this finding may have arisen because, in this condition, a phasic alerting together with a transient suppression of attention-orienting might have been elicited by the warning CC, followed by a reflexive orienting of attention to the stimulated space location as triggered by the target delivery.

As for the endogenous attention-orienting mode conditions, the alpha power recorded for LC task, where the cue was both alerting and validly informative of the spatial point at which the target would follow, showed to be somewhat lower than that elicited by CC condition over the LH, but not the RH, and, in general, pretty higher than that computed by FFT for the NC mode over both hemispheres. It seems highly plausible that alpha desynchronization found over the left hemisphere in response to LC condition may have undergirded a fast orienting of attention to the location indicated by the valid cue. A possibility strongly supported by Posner’s theses [44] who considered the left lateralized processes as more likely involved with higher temporal (phasic) mechanisms, such as orienting of attention, and right lateralized processes often more involved with slower (tonic) alerting effects. Consistent with these views, LC showed a same alpha power as CC over the RH, but a significant higher desynchronization (i.e., a significant lower alpha power) over the LH (see EEG alpha-power maps drawn in Figure 5c, and Figure 6 for mean power values for this condition). Interestingly, a right sided asymmetry in alpha scalp distribution has been shown by several previous studies [21,58].

Most interestingly, the data also indicated that introducing a neurocognitive perceptual (e.g., a target-type discrimination) and psychomotor overload (e.g., a double-choice motor task) as in the LCmot condition further increased alpha desynchronization over the LH, but not the RH. This left-sided increase in alpha desynchronization in LCmot with respect to both CC and LC showed to be of such a degree that the alpha for this cueing mode did show any differences from that for the exogenous NC task over the LH as compared to the RH. Conversely, LCmot elicited a same alpha power level as LC over the RH. As a result of the afore-mentioned changes, a prominent right-sided hemispheric asymmetry of the alpha power occurred for this motor-charged cueing-mode only (see EEG alpha-power maps drawn in Figure 5d, and Figure 6 for mean power values for this condition).

As for the functional meaning of a left-sided asymmetry of alpha desynchronization and a right-sided asymmetry of alpha synchronization found for LCmot cueing condition, it might be possible that, in line with Posner’s proposals [44], during this high-motor workload task the LH may have been engaged in the orienting of attention to the location indicated by the valid cue, as reflected by an increase of alpha desynchronization, but the demands of the target-type discrimination and of a decision relative to the double-choice overt response may have required to sustain alerting and/or attention over time, thus recruiting the slower, tonic processing by the RH, reflected at the scalp by an increase of alpha synchronization.

As far as hypoxia is concerned, our alpha measures indicated that this deficient-oxygenation condition mostly increased alpha power at right-sided brain posterior districts, and more in details striate and extrastriate occipital areas as well as parietal areas (Figure 7). However, some of our findings also indicated that hypoxia selectively enhanced alpha power at the left-sided anterior prefrontal areas (Figure 8). As for the effects of this respiratory condition on brain attentional processes, independent of brain areas involved, our results showed increases of alpha synchronization no matter the endogenous and exogenous nature of visuospatial attention orienting. Moreover, they also hinted at the view that hypoxia had diffused influences on all attentional processing networks (i.e., alerting, orienting and executive control networks), in that, no matter the network activated, hypoxia resulted in a general increase of alpha synchronization.

At least for the increase of alpha power in response to hypoxic hypoxia obtained at posterior occipital-parietal areas, our discoveries fully mesh with the findings reported by some previous studies (i.e., [27,30]). For truth sake, however, it must be said that these discoveries are only partially consistent with the results of another relatively recent study [32], and clearly in contrast with the results of some dated investigations (e.g., [28,29]). We believe that rather than to methodological and procedural differences in (1) the induction methods of hypoxia in neurologically healthy volunteers, (2) the different alpha frequency band ranges taken into account, and (3) the number of scalp sites at which the alpha power was computed, the different results obtained by the present investigation as compared to several previous studies (e.g., [28,29,34]) may be closely due to the participants’ neurocognitive functional state during EEG recordings. Indeed, while the latter studies analyzed the alpha power in the spontaneous EEG of volunteers who were not submitted to any active psycho-motor or attentional tasks, the investigations showing fully consistent findings with our own (i.e., [27,30]) measured alpha power in EEG recorded either during a presumably more environmentally-aware or attentional-driven condition—though not directly analyzed or accounted for—such as the eyes-open state, compared to the eyes-closed one [27] or during a simple, though somehow effortful, computerized visuospatial attentional task in which participants had to keep a moving target as much closer to the center of a screen denoted by a rectangle, in order to avoid the drifting of the target towards the edges of the screen [30].

Despite the lack of any report of the participants’ performance scores by these authors [30], it may be assumed that the aforementioned task might have been perceived as easier or less effortful in normoxia than in hypoxia, and that the generalized increases in alpha synchrony during the latter condition might have represented an alteration or, up-to a certain degree, a suppression of brain attentional orienting capacity. In support of this view, Zhao et al. [34] found changes of EEG during hypoxia/reoxygenation confined in posterior right cortices, with increased alpha synchrony related to hypoxia and increased beta desynchrony related to reoxygenation. Further support derived from our current data, which showed increased error rates and motor response times as well as of alpha power in hypoxia with respect to normoxia.

## 6. General Conclusions

All in all, the modulation of alpha power induced by the cueing conditions used in our study lends reliable and robust support to the view advanced by several previous studies e.g., [19,56,57] that the desynchronization versus the synchronization of this oscillatory-band reflects an active orienting versus an inhibition of visuospatial attention, respectively. Following this line of reasoning, the generalized right-sided alpha band synchronization observed in hypoxia as compared to normoxia would reflect the effort to sustain attention-alerting over time, as mediated by the RH, in order to cope with a transient impairment or, up to a certain degree, suppression of brain attention-orienting capacity, mediated by the LH. The lack of any effects of the different attentional cueing and executive conflict conditions on alpha synchronization induced by hypoxia may also indicate that this enhancement possibly reflected an overall transient alteration or tout-court suppression of attention alerting, orienting, and executive networks functionality independently from one another.

## 7. Study Limits and Wishes

A possible limitation of the present study was the relatively small sample size (*n* = 8.) The latter, in fact, was somewhat small and therefore conceivably statistically underpowered. However, the consistency of our general behavioral and electrophysiological findings with those of previous studies in the literature lends strong support to their soundness as also championed by partial eta squared values of computed size effects here provided. Furthermore, the sample size was large enough to assess the expected pattern of attentional effects predicted by available literature. Another edge for our data might be our use of parametric statistical tests which require the a-priori hypothesis of variables with normal distribution, here somehow not assured because of the lack of linearity of FFT values and of the small sample size. Nevertheless, because of the potential relevance of our findings, we think it is worth pursuing further research aimed at their sound replication by enlarging the sample size and the use of non-parametric statistical tests.
